# Essential Amino Acid Profile in Parenteral Nutrition Mixtures: Does It Meet Needs?

**DOI:** 10.3390/nu10121937

**Published:** 2018-12-06

**Authors:** Roberto Iacone, Clelia Scanzano, Lidia Santarpia, Lucia Alfonsi, Maurizio Marra, Maria Carmen Pagano, Anna D’Isanto, Ignazio Frangipane, Andrea Vitalone, Mariana D’Angeli, Franco Contaldo, Fabrizio Pasanisi

**Affiliations:** Internal Medicine and Clinical Nutrition, Department of Clinical Medicine and Surgery, “Federico II”, University Hospital, 80131 Naples, Italy; clelia.scanzano@unina.it (C.S.); lidia.santarpia@unina.it (L.S.); lucia.alfonsi@unina.it (L.A.); maurizio.marra@unina.it (M.M.); carmenpag@libero.it (M.C.P.); annadisanto85@hotmail.it (A.D.); frangipane.ignazio@gmail.com (I.F.); andreavitalone@libero.it (A.V.); dangelimariana@outlook.it (M.D.); franco.contaldo@unina.it (F.C.); pasanisi@unina.it (F.P.)

**Keywords:** parenteral nutrition, essential amino acids, branched-chain amino acids, leucine, isoleucine, methionine, whey protein

## Abstract

Background and Aims: The study compares the essential amino acid (EAA) composition of different parenteral nutrition (PN) mixtures with whey protein EAA profile and the theoretical daily EAA requirements (set by WHO/FAO/UNU or IAAO method). According to the individual EAA profile, the potential effect of several PN mixtures was evaluated on the skeletal muscle mass (SMM) of patients on home PN. Methods: Eight AA solutions and fifteen complete PN mixtures were considered. Twenty-nine clinically stable patients with short bowel syndrome on home total PN were retrospectively evaluated. SMM was estimated by bioelectrical impedance analysis. Results: The prescribed doses of EAA that showed a significant increase in home PN patients muscle mass were considerably greater than the theoretical ones, showing an EAA profile similar to whey protein. At the daily dose of 1 g of total AA s/kg body weight (BW), the considered PN mixtures mostly failed to improve SMM. Only prescribed doses which included more than 0.25 g/kg BW of total BCAA with at least 0.10 g/kg BW leucine, 0.08 g/kg BW isoleucine, and 0.06 g/kg BW methionine showed a significant increase in SMM. Conclusions: The theoretical daily requirement for each EAA was met by all considered PN solutions when the prescribed daily dose of total AAs was set at 1 g/kg BW. Nevertheless, our data suggest that only an increase in total BCAA, also richer in single AA leucine, isoleucine, and methionine, is associated with the maintenance and/or increase of SMM. According to these preliminary observations, we support the prescription of an EAA composition of PN mixtures close to that of whey protein for the preservation of SMM in patients on long-term total PN.

## 1. Introduction

Muscle wasting may occur in several conditions such as older age, long-term muscle disuse, malnutrition, cancer cachexia, other inflammatory and catabolic conditions [1,2]. In patients who cannot undergo oral feeding or enteral nutrition, it is necessary to provide nutrients intravenously (i.e., parenteral nutrition, PN), including amino acids (AAs) [3,4]. Generally, AA solutions for PN are defined “standard” if contain all nine essential AAs (EAAs), various nonessential amino acids (NEAAs) and some AAs considered conditionally essential [5,6]. However, commercially available standard AA solutions are quantitatively and qualitatively different in their EAA compositions. Furthermore, “specific” AA solutions could be required for certain clinical conditions, such as hepatic encephalopathy [7], AA disorders (e.g., inborn errors in AA metabolism [8]), neonatal intensive care [9] etc.

Despite the widespread use of PN both in the hospital and at home, studies on the clinical effectiveness of standard AA solutions with different EAA compositions appear dated or inadequate [10,11], and formulations of commercially available AA mixtures remained mostly unchanged since the 1980s. Indeed, the AA composition of standard AA solutions and of complete PN mixtures was designed to be similar to proteins of high biological value recommended for oral human nutrition (e.g., egg white, casein or soy) [12] to maintain a normal plasma amino acid pattern [13,14,15]. Nowadays, there is clear evidence that skeletal muscle mass (SMM) accretion requires an adequate amount of EAAs, mostly leucine, which triggers protein synthesis in skeletal muscle and plays a leading role in mammalian target of rapamycin complex 1 (mTORC1) activation [16]. Recently, it has been shown that oral administration of whey protein (a leucine-rich protein) is more effective than other food proteins [17,18,19,20] in promoting protein synthesis in skeletal muscle by improving muscle accretion and/or slowing down muscle mass loss in patients with wasting syndrome.

In light of the existing evidence, we compared the AA compositions of several currently available standard AA solutions and complete parenteral nutrition mixtures with that of whey protein. Furthermore, we evaluated whether the EAA daily requirement was met by the considered products while given at the recommended dose. The daily EAA requirements referenced in the present study were those set by the World Health Organization/Food and Agriculture Organization of the United Nations/United Nations University (WHO/FAO/UNU) [21] or by the indicator amino acid oxidation (IAAO) method [22]. Finally, the simple direct relationship between the received daily dose of total or individual EAAs or total branched-chain amino acids (BCAAs) and the change in SMM over time was retrospectively evaluated in twenty-nine clinically stable patients with short bowel syndrome (SBS) on total PN (the most accurate study method for assessing the amino acid and other nutrients intake).

## 2. Materials and Methods

### 2.1. AA Solutions and Complete Mixtures for Parenteral Nutrition

The AA composition of the proteins contained in whey, egg white, casein, and soy was obtained by the literature [18,19,20,21,22,23]. The AA compositions of eight standard AA solutions (Glamin^®^, Aminoven^®^, Sintamin^®^, Parentamin^®^ (Fresenius Kabi Italia, Isola della Scala, Italy), Amixal^®^, Freamine^®^ (BBraun, Milan, Italy), Isopuramin^®^, and Travasol^®^ (Baxter, Rome, Italy)) and of fifteen complete parenteral nutrition mixtures (Periven^®^, Kabiven^®^, Krinuven^®^, Smofkabiven^®^, Aminomix^®^ (Fresenius Kabi Italia, Isola della Scala, Italy), Nutriplus^®^, Nutrispecial^®^, Nutriperi^®^, Basalflex^®^, Periflex^®^, Plusflex^®^, Specialflex^®^ (BBraun, Milan, Italy), Clinimix^®^, OliClinomel^®^, Olimel^®^ (Baxter, Rome, Italy)) were obtained using technical sheets.

To compare daily EAA requirements with their content in the considered products, the daily dose of total AAs (TAAs) was hypothetically set at 1 g/kg body weight (BW). This allowed for comparison between different AA solutions while measuring their EAA content in mg per gram of total AA. In the event that the prescribed daily dose of TAAs was different from 1 g/kg BW, the EAAs values reported for the considered products have to be multiplied by the actual prescribed dose.

### 2.2. Patients

Twenty-nine SBS patients (12 males, 17 females) on home total PN for at least 30 days, and in stable clinical, metabolic and nutritional conditions were retrospectively evaluated; follow-up evaluations were conducted at the Clinical Nutrition Unit of Federico II University Hospital Naples in 2017. Body weight, height, parenteral mixture composition, length of treatment and patient history were collected from electronic health records. SMM evaluations were carried out at entry and follow-up patient’s visit. For each patient there were several SMM evaluations and changes in the AA prescribed dose. We selected pairs of measurements in a time range where the doses prescribed in PN mixtures were constant. Thus, two SMM evaluations at two different time points were considered for all patients. SMM was estimated by bioelectrical impedance analysis (BIA) using the Tengvall [24] equation including height (cm), body weight (kg), resistance (ohm), reactance (ohm) and sex: SMM (kg) = −24.02 + (0.33 × height) + (−0.031 × resistance) + (0.083 × reactance) + (0.046 × body weight) − 1.58 × (0 if male, 1 if female). BIA measurements were carried out early in the morning, after interrupting PN at midnight, in a thermoneutral environment (25–26 °C) by the same operator using the analyzer Human IM-Plus II; DS Medica, Monza, Italy. The SMM change over time (delta SMM) was calculated by subtracting the value of the first measurement to the second. For all patients, the composition of parenteral mixtures was kept constant between the first and the second BIA measurement. Fourteen patients received a standard, industrially manufactured parenteral nutrition mixture, whereas fifteen patients had a compounded personalized mixture. The amino acid solution in the compounding of personalized mixtures was Sintamin^®^ 10% (in 13 patients) or Glamin^®^ 13.4% (in 2 patients).

### 2.3. Statistical Analysis

Statistical analyses were performed using the Statistical Package for the Social Sciences (IBM SPSS Statistics for Windows, Version 22.0, IBM Corp.: Armonk, NY, USA). Nonparametric partial correlation analyses (Spearman’s rank-order correlation) were used to estimate the relationship between the analyzed variables. The one-way ANCOVA (analysis of covariance) was performed to assess differences between groups on a single dependent variable after controlling for the effects of more covariates. A value of *p* < 0.05 was considered statistically significant.

## 3. Results

### 3.1. AA Solutions and Complete Mixtures for Parenteral Nutrition

**Figure 1:**
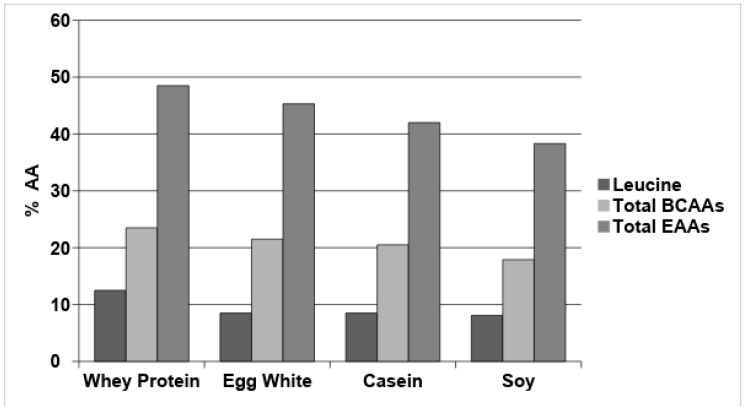
The leucine, total branched-chain amino acids (BCAAs) and total essential amino acids (EAAs) content of whey protein compared with that of egg white, casein and soy protein.

Figure 1 shows that whey protein has a higher leucine, total BCAAs, and EAAs content, compared with egg white, casein, and soy proteins, traditionally considered the gold standard of food proteins. The AA solutions and complete nutrition mixtures evaluated included all nine EAAs and a variable NEAAs composition ranging from 25% (Isopuramin^®^) to 59% (Amixal^®^), and from 49% (Periven^®^, Kabiven^®^, Olimel^®^) to 57% (Krinuven^®^, Smofkabiven^®^, Aminomix^®^) in pure AA solutions and complete parenteral mixtures, respectively.

Table 1 shows the daily EAA requirement for adults (2007 WHO/FAO/UNU and IAAO method) [21,22], the total amounts of EAAs and NEAAs in whey protein and the total amounts of EAAs and NEAAs in the main, commercially available, intravenous standard AA solutions. When the prescribed daily dose of TAAs was 1 g/kg BW, all solutions met the requirements: (a) for each EAA from 1.0- to 8.0 fold; (b) for total BCAAs from 1.8- to 3.6 fold, and (c) for total EAAs from 2.2- to 4.1 fold. In the event that the prescribed daily dose of TAAs with intravenous standard AA solutions was different from 1 g/kg BW, the EAAs values reported in table 1 for intravenous standard AA solutions have to be multiplied by the actual administered dose. When each EAA of whey protein was set at 100%, the leucine content ranged from 46% (Glamin^®^) to 94% (Parentamin^®^); the total amount of BCAAs ranged from 65% (Glamin^®^) to 129% (Isopuramin^®^); and the total amount of EAAs ranged from 74% (Amixal^®^) to 134% (Isopuramin^®^).

Table 2 shows the daily EAA requirements for adults (2007 WHO/FAO/UNU and IAAO’s method), the total amounts of EAAs and NEAAs in whey protein and the total amounts of EAAs and NEAAs in the main commercially available complete mixtures. When the prescribed daily dose of TAAs was 1 g/kg BW, all considered products met the requirements for each EAA from 1.6- to 6.0 fold, for total BCAAs from 2.2- to 2.4 fold and for total EAAs from 2.4- to 2.8 fold. When the prescribed daily dose of TAAs with complete parenteral nutrition mixtures was different from 1 g/kg BW, the EAAs values reported in Table 2 for complete mixtures have to be multiplied by the actual administered dose. In the complete mixtures, when each EAA of whey protein was set at 100%, the leucine content ranged from 55% (Olimel^®^) to 62% (Nutriplus^®^, Nutrispecial^®^, Nutriperi^®^, Basalflex^®^, Periflex^®^, Plusflex^®^, Specialflex^®^); the total BCCAs ranged from 78% (Periven^®^, Kabiven^®^, Olimel^®^) to 86% (Nutriplus^®^, Nutrispecial^®^, Nutriperi^®^, Basalflex^®^, Periflex^®^, Plusflex^®^, Specialflex^®^); and the total amount of EAAs ranged from 77% (Krinuven^®^, Smofkabiven^®^, Aminomix^®^) to 91% (Periven^®^, Kabiven^®^, Olimel^®^).

### 3.2. Patients

**Figure 2:**
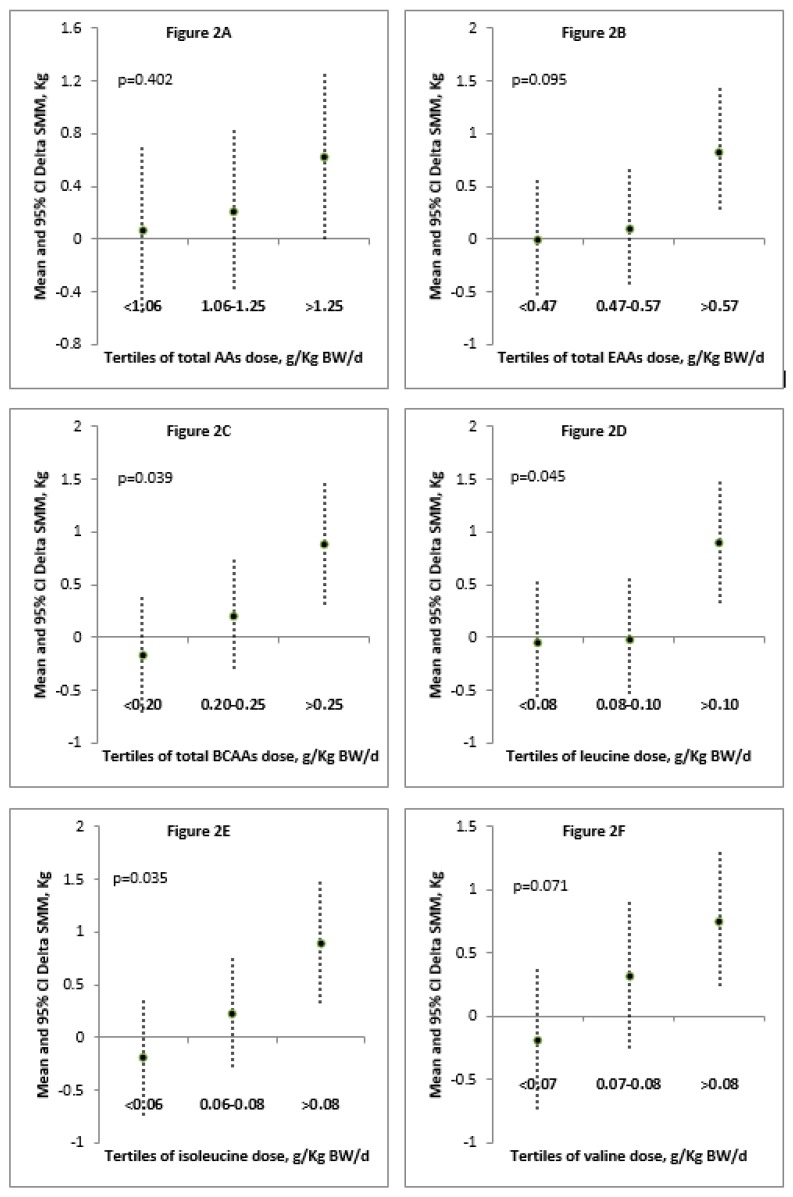

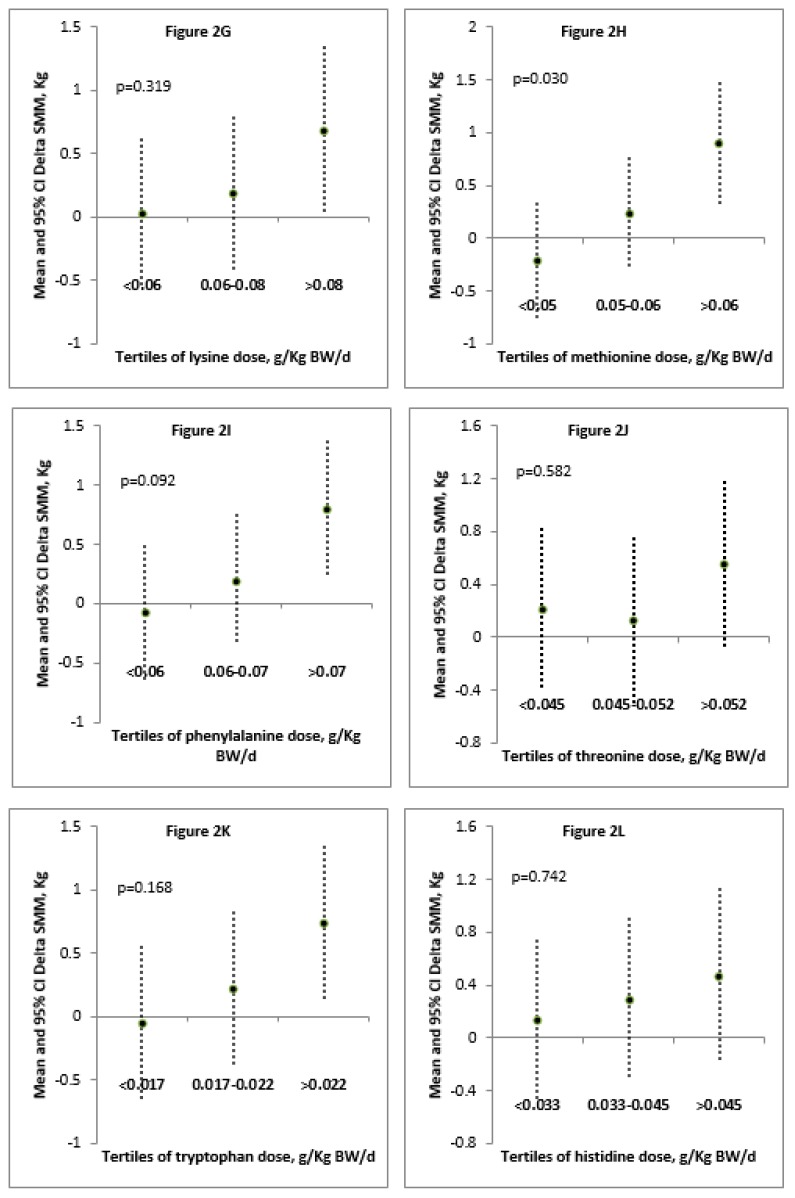
Mean and 95% confidence interval (CI) of delta SMM compared to the tertiles of prescribed doses of TAAs, total EAAs, total BCAAs or individual EAAs. The differences between the change in muscle mass over time (delta SMM) and the prescribed doses of AAs were evaluated by analysis of covariance controlling for age, gender and interval between the two SMM measurements.

Table 3 lists demographic features of the twenty-nine SBS patients on home long-term total PN and the prescribed doses of TAAs, EAAs, BCAAs and of each EAA per kg BW in the parenteral mixtures. The difference in the doses per kg BW of each EAA depends on the different AA composition of the administered products, the prescribed dose of TAAs and the BW of the patient. In Table 4, age, gender, and time adjusted nonparametric Spearman’s analysis indicated a significant positive correlation between delta SMM and prescribed daily doses per kg BW of total EAAs, total BCAAs, single AAs leucine, isoleucine, valine, and methionine with PN products to the twenty-nine SBS patients. No significant correlation was found between delta SMM and daily doses per kg BW of TAAs or other EAAs considered. The time between the two measurements of SMM ranged from 30 to 356 days (delta time). Figure 2A,L, depict the mean and 95% confidence interval of delta SMM compared to the tertiles of prescribed doses of total EAAs, total BCAAs or individual EAAs. Analysis of covariance between these tertiles and delta SMM (controlling for age, gender and interval between the two SMM BIA measurements) highlights that only prescribed doses which included more than 0.25 g/kg BW of total BCAA (*p* = 0.039) with at least 0.10 g/kg BW of leucine (*p* = 0.045), 0.08 g/kg BW of isoleucine (*p* = 0.035), and 0.06 g/kg BW of methionine (*p* = 0.030) allowed a significant increase in SMM during the time of observation.

As showed in Figure 2A,L, the mean of Delta SMM between the first two tertiles did not differ statistically from each other (in fact, the two 95% CI were always quite overlapping) and it was sometimes negative (only for the first tertile), zero or slightly above zero (for the second tertile). In the third tertile of doses, the mean of delta SMM was always well above zero and (for BCAA-Figure 2C, leucine-Figure 2D, isoleucine-Figure 2E, and methionine-Figure 2H) was significantly different from the ones of lower tertiles. In other words, for patients in the first two tertiles of doses, Delta SMM may be reduced, zero or slightly increased. Only for the third tertile of doses there was a remarkable increase in Delta SMM (95% CI never touched the *x*-axis). A zero increase/decrease of SMM, i.e., maintenance of muscle mass, occurred in the range of doses of the second tertile. However, a statistical difference was not reached between the first and second tertiles. As a consequence, the study was unable to distinguish between the AA dose to maintain or decrease muscle mass.

## 4. Discussion

This study specifically addresses the essential AA composition of standard intravenous AA solutions and complete PN mixtures commercially available in our Country. Evaluations of AAs that are considered conditionally essential (i.e., glutamine, arginine, cysteine, and taurine) have been recently addressed [5,6]. Our clinical observations examined adult patients on home total PN under stable clinical, metabolic, and nutritional conditions whose EAA requirement is close to the one expected for the adult population.

It is widely acknowledged that intravenous infusion of mixtures containing all nine EAAs and a variable amount of NEAAs is necessary to limit muscle mass loss, promote muscle protein synthesis and preserve nitrogen balance [1,3,4] in patients on PN. However, due to the lack of controlled clinical trials on the effect of different AA formulations on the clinical outcome, we mostly rely on uncontrolled studies or dedicated guidelines [5,10,11]. These dedicated guidelines provide information on the recommended total daily amount of AAs, but not on the specific AA composition; guidelines also presume the same daily AA requirement for patients on both total parenteral or enteral nutrition [1,2,3,4,5,6,7,8]. Recent studies on muscle metabolism have suggested that the AA composition of whey protein is more effective than that of other food proteins in triggering muscle protein synthesis and reducing protein breakdown in skeletal muscle [18]. These studies showed that positive effects are strongly related to whey protein’s higher content of EAAs and BCAAs, in particular leucine [19,20,21,22,23,24,25]; conversely, the positive effects are not related to the digestibility or absorption rate of AAs derived from such proteins, as initially thought [19]. It is well documented that leucine acts as a substrate for muscle protein synthesis and works as a trigger for protein synthesis in skeletal muscle by activating mTORC1 signaling [16]. Despite these new insights, commercially available parenteral AA formulations are based on the composition of food proteins traditionally considered the gold standard (egg white, casein, and soy) [15] or on studies of the plasma amino acid profile of normal subjects [12,13,14]. The standard AA formulations evaluated in this study slightly differed from each other in composition; we studied solutions with different concentrations of specific EAAs as well as with different amounts and types of NEAAs. When supplied at the usually prescribed dose of 1 g of total AA/kg BW, all considered AA solutions met the healthy adult’s requirements for each EAA as recommended by 2007 WHO/FAO/UNU report or by the IAAO method. Moreover, the studied solutions for single EAA exceeded the requirements up to 8-fold. On the other hand, leucine content of the AA solutions was generally less than that of whey protein, reaching 94% in one solution (Parentamin) only. Complete AA mixtures for parenteral nutrition achieved a maximum of 62% of the recommended leucine content (Nutriplus, Nutrispecial, Nutriperi, Basalflex, Periflex, Plusflex, Specialflex).

Even considering the limits of a preliminary and retrospective clinical observation, we observed that muscle mass gain in the patients on home PN was significantly and positively associated with the daily doses of total EAAs, of BCAAs and of single EAA leucine, isoleucine, valine, and methionine; this gain was not associated with other EAAs or TAAs content. Overall, our results seem consistent with the observations of Volpi E et al. [26], whose experiments showed that NEAAs were unnecessary for the stimulation of muscle protein anabolism and independent from the TAAs content of the nutritional mixtures. However, this latter point seems to be contradicted by some dietetic experiments on the protein content of the meal and post-prandial muscle protein synthesis (MPS) showing a direct relation between MPS and the amount of the protein in the experimental meal [27]. Furthermore, our data highlight a strong correlation between the amount of leucine and isoleucine in the solutions and the SMM gain. This is in agreement with the pivotal function of leucine which triggers protein synthesis in skeletal muscle mass and the recent studies by Zhang S et al. [28] suggesting that isoleucine supplementation enhances muscular glucose transporter expression, which could have important implications for muscle growth. Regarding methionine, it is the most common start codon in the standard genetic code. In fact, protein synthesis is universally initiated with the amino acid methionine although it is usually removed by post-translational modification.The significant association between the dose of methionine in the PN mixtures and muscle restoration seems reasonable.

The authors have no explanation why delta SMM is not significantly correlated with all individual EAAs. It could only be speculated that some EAAs also have the pivotal role to trigger and promote muscle protein synthesis, besides to be a substrate like others EAAs.

The study has some limitations. The first limitation is that the study is uncontrolled and not specifically designed (it is a retrospective study). The second and perhaps most important limitation concerns the small number of patients. For these reasons, the reported doses of AAs showing a significant increase in SMM are to be considered only indicative. Well designed and controlled studies, taking into account the gender and the initial muscle mass, are requiredto define the right dose of EAA to preserve or gain SMM in long-term PN patients.

## 5. Conclusions

The daily requirement of each EAA, as established by the WHO/FAO/UNU [20] or the IAAO method [22], is met by all considered products when the prescribed dose of TAAs is set at 1 g/kg BW/day. However, the dose of leucine, isoleucine, and methionine, in particular in the mixtures for PN, appear not to be enough to trigger an anabolic response and therefore muscle growth. Actually, at the usual prescribed doses, the currently available nutritional parenteral mixtures seem to have less essential AAs than necessary to obtain positive effects on muscle mass preservation or gain. Consequently, in order obtain the desirable doses of total BCAAs, leucine, isoleucine, and methionine, a higher dose of TAAs could be necessary. This finding, according to our preliminary observations, suggests testing new AA formulations containing higher amounts of BCAAs, leucine, isoleucine, and methionine close to whey protein AAs profile (possibly without the unnecessary renal load of non-essential amino acids). Further investigation is required to assess the possible beneficial effect on SMM in patients on long-term total PN at risk of muscle wasting and sarcopenia.

## Figures and Tables

**Table 1:** Adult daily EAA requirement (mg/kg body weight (BW)/day (d)); EAAs and total nonessential amino acids (NEAAs) content in whey protein and in commercially available IV standard amino acid solutions (mg EAA/g TAAs).

EAA (mg)	mg EAA/kg BW/d		mg EAA/g TAAs								
	Adult EAA Requirement ^a^		Whey Protein	Commercially Available IV Standard Amino Acid Solutions							
	WHO/FAO/UNU (2007 Report)	IAAO Method		Glamin^®^ 13.4%	Aminoven^®^ 10%	Sintamin^®^ 10%	Isopuramin^®^ 10%	Parentamin^®^ 10%	Amixal^®^10%	Freamine^®^10%	Travasol^®^10%
Leucine	39	-	125	58	74	95	106	118	89	94	73
Isoleucine	20	-	60	41	50	73	80	55	50	71	60
Valine	26	-	50	53	62	69	116	97	62	68	58
Total BCAAs	85	144	235	152	186	237	302	270	201	233	191
Lysine	30	36	105	66	66	77	115	153	28	75	58
Methionine + Cysteine	15	12.6	55	41	43	57	73	28	44	57	40
Threonine	15	19	45	41	44	42	95	58	42	41	42
Histidine	10	-	20	50	30	35	50	21	30	29	48
Phenylalanine + Tyrosine	25	42	75	60	55	60	82	37	51	58	96
Tryptophan	4	4	25	14	20	16	32	17	16	15	18
Total EAAs	184	250	560	424	444	524	749	584	412	508	493
Total NEAAs	-	-	440	576	556	476	251	416	588	492	507

AA: amino acid; TAAs: total amino acids; EAA: essential amino acid; NEAA: nonessential amino acid; BCAA: branched-chain amino acid; IV: intravenous; WHO/FAO/UNU: World Health Organization/Food and Agriculture Organization of the United Nations/United Nations University; IAAO: Indicator Amino Acid Oxidation. ^a^ In order compare the daily EAA requirement with the content of EAAs in the considered products, the daily dose of TAAs was hypothetically set at 1 g/kg BW. This allowed for comparisons between different AA solutions while measuring the EAA content in mg per gram of TAAs. In the event that the daily dose of TAAs supplied with IV standard AA solutions was different from 1 g/kg BW, the EAAs values reported for IV standard AA solutions have to be multiplied by the actual administered dose.

**Table 2:** Adult daily EAA requirement (mg/kg body weight (BW)/day (d)); EAAs and total NEAAs content in whey protein and in commercially available complete parenteral mixtures (mg EAA/g TAAs).

EAA (mg)	mg EAA/Kg BW/d		mg EAA/g TAAs					
	Adult EAA Requirement ^a^			Commercially Available IV Complete Parenteral Mixtures				
	WHO/FAO/UNU (2007 Report)	IAAO Method	Whey Protein	Periven^®^ Kabiven^®^	Krinuven^®^ Smofkabiven^®^ Aminomix^®^	Nutriplus^®^ Nutrispecial^®^ Nutriperi^®^ Basalflex^®^ Periflex^®^ Plusflex^®^ Specialflex^®^	Clinimix^®^ Oliclinomel^®^	Olimel^®^
Leucine	39	-	125	71	76	78	73	69
Isoleucine	20	-	60	49	52	59	60	50
Valine	26	-	50	64	62	65	58	64
Total BCAAs	85	144	235	184	190	202	191	183
Lysine	30	36	105	80	49	57	58	79
Methionine + Cysteine	15	12.6	55	49	43	49	40	50
Threonine	15	19	45	49	45	45	42	50
Histidine	10	-	20	60	30	31	48	60
Phenylalanine + Tyrosine	25	42	75	73	55	88	60	72
Tryptophan	4	4	25	17	20	14	18	17
Total EAAs	184	250	560	512	433	486	457	511
Total NEAAs	-	-	440	488	567	514	543	489

AA: amino acid; TAAs: total amino acids; EAA: essential amino acid; NEAA: nonessential amino acid; BCAA: branched-chain amino acid; IV: intravenous; WHO/FAO/UNU: World Health Organization/Food and Agriculture Organization of the United Nations/United Nations University; IAAO: Indicator Amino Acid Oxidation. ^a^ In order compare the daily EAA requirement with the content of EAAs in the considered products, the daily dose of TAAs was hypothetically set at 1 g/kg BW. This allowed for comparisons between different complete parenteral nutrition mixtures while measuring the EAA content in mg per gram of TAAs. In the event that the daily dose of TAAs supplied with complete parenteral nutrition mixtures was different from 1 g/kg BW, the EAAs values reported for complete parenteral nutrition mixtures have to be multiplied by the actual administered dose.

**Table 3:** Age, weight, height, change in skeletal muscle mass (delta SMM) over time, time course between the two SMM measurements (delta time) and prescribed daily amino acid doses in g per kg body weight (BW) to twenty-nine patients (12m/17f) on home total parenteral nutrition in stable clinical conditions. Mean ± SD and minimum-maximum (min-max) values.

	Mean ± SD	Min-Max
Age (y)	49 ± 17	24-75
Weight (kg)	55 ± 12	42-81
Height (cm)	162 ± 10	141-182
Delta SMM (kg)	0.3 ± 0.9	−1.5-2.2
Delta Time (days)	143 ± 94	30-356
TAAs/kg BW/day (g)	1.15 ± 0.36	0.53-1.98
EAAs/kg BW/day (g)	0.54 ± 0.17	0.28-0.98
BCAAs/kg BW/day(g)	0.24 ± 0.08	0.13-0.44
Leucine/kg BW/day (g)	0.09 ± 0.03	0.05-0.18
Isoleucine/kg BW/day (g)	0.07 ± 0.02	0.04-0.14
Valine/kg BW/day (g)	0.07 ± 0.02	0.04-0.13
Lysine/kg BW/day (g)	0.07 ± 0.02	0.04-0.14
Methionine/kg BW/day (g)	0.06 ± 0.02	0.03-0.11
Phenylalanine/kg BW/day (g)	0.07 ± 0.03	0.03-0.15
Threonine/kg BW/day (g)	0.05 ± 0.02	0.02-0.09
Tryptophan/kg BW/day (g)	0.02 ± 0.01	0.01-0.04
Histidine/kg BW/day (g)	0.04±0.01	0.02-0.07

BW = body weight; TAAs = total amino acids; EAAs = total essential amino acids; BCAAs = total branched chain amino acids.

**Table 4:** Non parametric correlations (rho of spearman) between the change in skeletal muscle mass (SMM) in Kg and prescribed doses (daily g per Kg body weight) of total amino acids (TAAs), EAAs, BCAAs, leucine, isoleucine, valine, lysine, methionine, phenylalanine, threonine, tryptophan, and histidine to twenty-nine patients (12m/17f) on home long-term total parenteral nutrition. Rho of spearman and corresponding p were calculated controlling for gender, age and the interval between the two measures of SMM (delta time). Delta time between the two measurements of SMM ranged from 30 to 356 days.

Correlation between Delta SMM (kg) and Daily Dose (g) of:	rho	*p*
TAAs/kg BW/day	0.350	0.080
EAAs/kg BW/day	0.398	0.044 *
BCAAs/kg BW/day	0.460	0.018 *
Leucine/kg BW/day	0.463	0.017 *
Isoleucine/kg BW/day	0.434	0.027 *
Valine/kg BW/day	0.439	0.025 *
Lysine/kg BW/day	0.348	0.081
Methionine/kg BW/day	0.447	0.022 *
Phenylalanine/kg BW/day	0.359	0.072
Threonine/kg BW/day	0.311	0.122
Tryptophan/kg BW/day	0.338	0.091
Histidine/kg BW/day	0.258	0.204

* = significant correlation; BW= body weight; TAAs = total amino acids; EAAs = total essential amino acids; BCAAs = total branched chain amino acids.

## Abbreviations

AA	amino acid
BCAA	branched-chain amino acid
BIA	bioelectrical impedance analysis
BW	body weight
EAA	essential amino acid
FAO	Food and Agriculture Organization of the United Nations
IAAO	indicator amino acid oxidation
mTORC1	mammalian target of rapamycin complex 1
NEAA	nonessential amino acid
PN	parenteral nutrition
SBS	short bowel syndrome
SMM	skeletal muscle mass
TAA	total amino acid
UNU	United Nations University
WHO	World Health Organization
